# Links between intuitive and mindful eating and mood: do food intake and exercise mediate this association?

**DOI:** 10.3389/fnut.2025.1458082

**Published:** 2025-05-30

**Authors:** Murat Açik, Nazlı Nur Aslan Çin

**Affiliations:** ^1^Faculty of Health Sciences, Department of Nutrition and Dietetics, Fırat University, Elâzığ, Türkiye; ^2^Faculty of Health Sciences, Department of Nutrition and Dietetics, Karadeniz Technical University, Trabzon, Türkiye

**Keywords:** intuitive eating, mindful eating, psychological distress, dietary patterns, exercise

## Abstract

**Background:**

In recent years, intuitive eating (IE) and mindful eating (ME) have emerged as promising approaches for developing a healthier relationship with food. Research also suggests that diet can significantly impact one's mental health. Nevertheless, there is no evidence to suggest that IE or ME can be associated with mental wellbeing through the influence of dietary patterns or practices. This study investigates the influence of IE and ME on mental wellbeing by affecting dietary practices.

**Methods:**

In this cross-sectional study, 990 adults were administered the intuitive eating scale-2 (IES-2), the mindful eating questionnaire (MEQ), and the Food Mood Questionnaire (FMQ). Briefly, FMQ consists of weekly servings of food groups known to influence brain function and chemistry and the Kessler psychological distress scale (K-6). Correlation analyses were performed to determine the relationships among K-6, IES-2, MEQ, and food groups, and path analyses were performed to assess the mediating effects of food groups on the relationship between the K-6 scale, IES-2, and MEQ.

**Results:**

A moderate negative association was found between the K-6 scale and IES-2 and MEQ (r = −0.47 and −0.36, respectively). Furthermore, exercise, breakfast, dairy products, fruits, and dark green leafy vegetables (DGLV) were directly related to psychological distress (path coefficient = −0.104, −0.151, −0.181, −0.235, and −0.238, respectively), while IES-2 and MEQ had indirect associations with psychological distress via FMQ components (path coefficient = −0.262 and −0.212). Among the IES-2 sub-dimensions, Unconditional Permission to Eat (UPE) and Reliance on Hunger and Satiety Cues (RHSC) were found to be indirectly associated with psychological distress through mediatory effects of exercise, breakfast, dairy, fruit, and DGLV. Breakfast and DGLV also mediated partially indirect negative effects of recognition (a sub-dimension of MEQ) on the K-6 scale.

**Conclusion:**

This study showed that adopting IE and ME habits effectively associated dietary and lifestyle practices and that some dietary practices and exercise were important for the link between IE and ME to mental distress.

## 1 Introduction

The act of eating is a fundamental biological process that enables an individual to survive and maintain homeostasis. However, various genetic, hormonal, social, and cultural factors can drive energy intake even in the absence of physiological need (1). In recent years, two novel behavioral approaches have emerged that focus on the physiological eating process by reducing the influence of external factors on energy intake. One of these is the concept of mindful eating (ME) and intuitive eating (IE) (2).

The ME and IE are two distinct yet complementary approaches to eating behavior that emphasize internal cues and awareness. They were developed to address disordered eating and the psychological determinants of overeating (3). The high level of overlap between IE and ME has led to the two concepts being used together or interchangeably in both research and literature reviews. However, it has been suggested that it would be more accurate to analyze IE and ME separately to gain a full understanding of the intrinsic eating style (4).

ME involves paying close attention to the sensory and experiential aspects of food consumption without aiming to lose weight or restrict intake (5). In contrast, IE focuses on recognizing and responding to bodily signals, promoting a healthy relationship with food by dismantling restrictive food rules and fostering self-acceptance (6). IE encourages eating based on physiological cues of hunger and satiety rather than external stimuli such as emotions or food availability (7). Indeed, the concept of IE is predicated upon the establishment of trust in the body, the cultivation of a positive relationship with food, and the prioritization of the inner self over external advice (8). While both approaches aim to improve eating behaviors, IE emphasizes emotional and instinctual connections to food, whereas ME prioritizes present-moment awareness and sensory engagement (9).

A variety of scales are employed for IE. The most commonly employed of these is the intuitive eating scale (IES)-2 scale (10). The scale comprises four subscales. The scale comprises four subscales: unconditional food permission (UPE), eating for physical rather than emotional reasons (EPR), reliance on hunger and satiety cues (RHSC), and body food choice congruence (B-FCC). UPE reflects an individual's willingness to consume food when hungry and a refusal to prohibit certain foods. EPR indicates that an individual eats for physical hunger without external stimuli. RHSC measures the extent to which an individual relies on hunger and satiety cues to guide their behavior. B-FCC includes the concept of nutrition-promoting taste preferences and nutrient choices that honor health and functionality (11).

Recent studies have identified IE as a promising approach for eliminating the maladaptive and self-sabotaging dieting mindset triggered by dietary interventions and for establishing a healthier relationship with food (12, 13). Nevertheless, two studies have yielded conflicting results in a large sample regarding the subscales of IE and dietary intake, energy intake, and the quality of diet consumed (11, 14). Previous research examining the relationship between IE and dietary intake has yielded inconsistent results (15–17). To date, only two studies have employed the subscales of the IES-2 scale in a general sample. This is because the scale is typically applied to specific populations, such as women, university students, young adults, and middle-aged adults (14). Horwath et al. (11) found that UPE was positively associated with a higher frequency of consumption of salty snacks, sweets, meat, and fast food and a lower frequency of vegetable, fruit, and whole grain intake. However, in the systematic review evaluating IE and ME, it was concluded that the interventions lacked participant diversity, focused on clinical populations and that these interventions did not affect dietary intake or quality (3, 18). Therefore, evaluating the association of IE and ME with dietary intake in the general population may help to fill the gap in the literature.

In recent years, an increasing number of individuals from a diverse range of social backgrounds have been experiencing a wide spectrum of mental health issues. According to data from the World Health Organization (WHO), in 2019, there were 264 million people experiencing depression, 45 million with bipolar disorder, 50 million with dementia, and 20 million with schizophrenia (19). Individuals' mental health status can be affected by lifestyle changes such as exercise and food intake (20). One of the most significant variables influencing mood in the therapeutic approach is dietary intake. It has been demonstrated that certain dietary choices can significantly impact an individual's mood (21). Various dietary rules and patterns may increase mental distress by altering the levels or receptor activities of neurotransmitters in the brain. Malnutrition or an unbalanced diet is therefore associated with the underlying pathophysiology of mental distress (22). For instance, the consumption of nutritionally dense foods such as vegetables and fruits has been demonstrated to positively influence mood and mental health (23). A systematic review of the literature has revealed that a healthy diet, regular exercise, and breakfast consumption have a positive association with mental health (24–26). Moreover, approaches like ME and IE promote awareness of food choices, leading to healthier eating behaviors and reduced emotional stress. These practices have shown positive outcomes in managing stress and improving overall mental wellbeing by fostering a better relationship with food (27). Nevertheless, the impact of IE or ME on mental distress by influencing dietary patterns or dietary practices has not been previously demonstrated. This study aims to investigate the association of IE and ME with mental distress by influencing dietary intake. It was hypothesized that: (1) IE would be inversely associated with mental distress; (2) ME would be inversely associated with mental distress; and (3) dietary patterns and exercise would be expected to mediate the relationship between these positive eating behaviors (IE and ME) and psychological distress. To the best of our knowledge, this is the first study to evaluate the association of dietary intake and exercise on the impact of IE and ME on mental distress.

## 2 Methods

### 2.1 Participants

This is a cross-sectional analytical study, conducted in the two Nutrition and Diet Counseling Centers in Elazig/Türkiye. Data was collected from April to June 2024. Adult participants aged between 20 and 64 were recruited after completing the informed consent form. Eligible participants were literate, non-pregnant or not in the lactation period, and had no serious illness. Participants with eating disorders, persons with alcohol abuse, individuals with kidney diseases, recurrent hypoglycemia, patients on medication or appetite-altering treatments (e.g., bariatric surgery), and psychiatric patients were excluded. We also excluded neurological patients who were unable to communicate. The University of Firat Institutional Review Board approved the study, and participants provided consent by opting into the survey.

Efforts were made to minimize response bias, particularly social desirability bias, given the self-reported nature of the scales used. Participants were informed that their responses would remain completely anonymous and confidential. Furthermore, the questionnaire was self-administered in a private setting to reduce external influence.

### 2.2 Calculation of sample size and procedure

The sample size was calculated using G^*^Power version 3.1.9.6. The study's power (1–β) was set at 0.90, with a Type I error rate (α) of 0.05. Based on a prior study that reported a linear relationship between IE and psychological stress [β regression coefficient (SE) = −2.83 (1.17), *t*-value = −2.42, *p*-value = 0.018] (27) the required sample size was determined to be 219 participants. To enhance statistical power for structural equation modeling (SEM), data collection exceeded this minimum.

**Figure 1:**
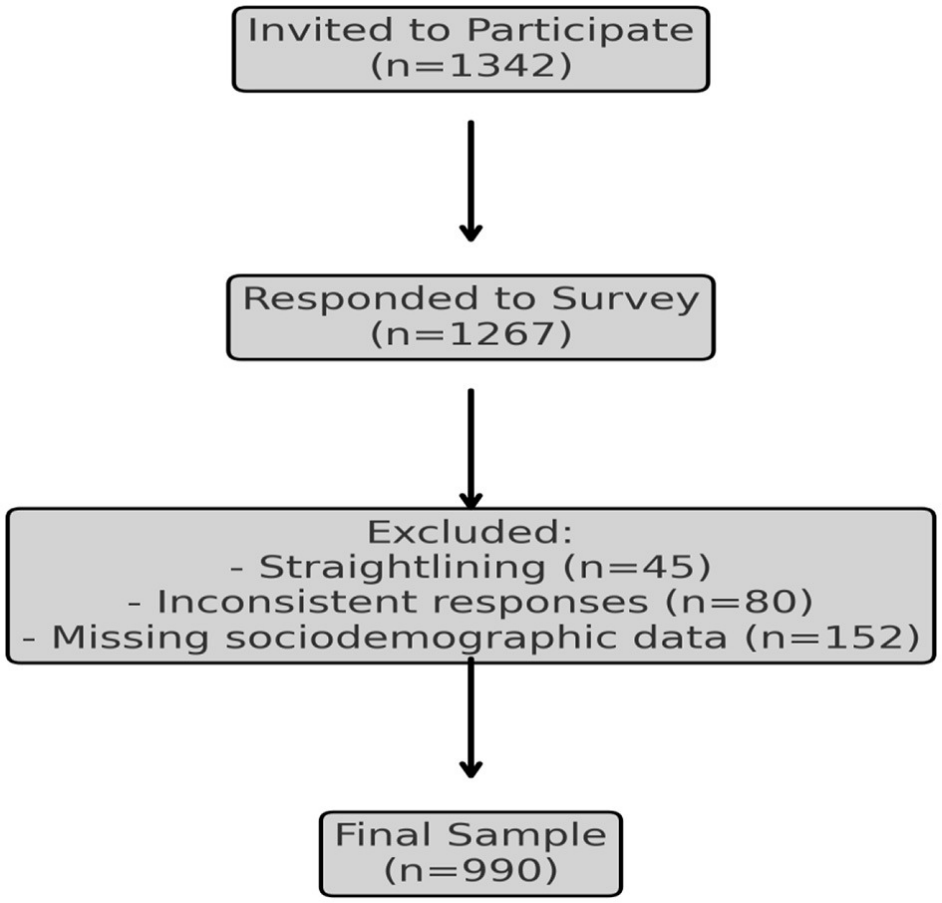
CONSORT diagram of participant flow.

Of the 1,342 individuals invited to participate, 1,267 provided responses. Participants who demonstrated straight lining behavior (i.e., repeatedly selecting the same response option across survey scales) or displayed inconsistencies within scale responses were excluded. Additionally, participants with missing data on key sociodemographic variables (age and gender) were removed from the analysis. Following these exclusions, the final analytical sample consisted of 990 participants. The diagram illustrating the flow of participants through the study is provided in Figure 1.

### 2.3 Measures

#### 2.3.1 Demographic data and health characteristics

Data regarding participants' gender, age, region of residence, education status, smoking status, status of chronic diseases, and drug use was collected via the questionnaire. Participants reported their current weight and height, which were used to calculate their body mass (kg/m^2^).

#### 2.3.2 Intuitive eating scale

IE was assessed using the IES-2, which includes 23 items scored on a 5-point Likert scale from 1 (strongly disagree) to 5 (strongly agree) and distributed across four subscales: UPE, EPR, RHSC, and B-FCC. Subscale scores were calculated by averaging responses, and the total IES-2 score was computed as the mean of all items after reverse scoring negatively phrased items (10). The IES-2 has demonstrated good psychometric properties, including internal consistency, test-retest reliability, and construct validity, in previous studies (28). In the present study, internal consistency was evaluated, yielding a Cronbach's alpha of 0.88 for the total scale and ranging from 0.76 to 0.91 for the subscales, indicating acceptable to excellent reliability.

#### 2.3.3 Mindful eating questionnaire

The abbreviated 20-item, two-factor MEQ was utilized to assess ME. This questionnaire comprises two factors: (1) Awareness—eating while being conscious of physiological and psychological experiences, and (2) Recognition—eating with consideration of hunger and satiety cues. Each item is rated on a 4-point Likert scale ranging from never/rarely to usually/always. The awareness component consists of 11 items, while the recognition component includes 9 items. Higher scores indicate a greater level of ME (29). In Türkiye, two items were removed and revised during the scale's validation process. For the modified 18-item version of the MEQ, the Cronbach's alpha coefficients were as follows: Awareness (α = 0.84) and Recognition (α = 0.78), demonstrating acceptable internal consistency (30).

#### 2.3.4 Food-mood questionnaire

Dietary consumption patterns were evaluated using a validated FMQ (31). The FMQ evaluates weekly servings of food groups known to influence brain function and chemistry, including whole grains, dairy, caffeine, fruits, nuts, high glycemic index (HGI) foods (e.g., rice and pasta), meat, vegetables, beans, fish, and fast food. Frequency of breakfast consumption, exercise (assessed with the question, “In the past 7 days, on how many days did you engage in at least 20 min of exercise?” with response options ranging from 0 days to more than 4 days), and use of multivitamins and fish oil supplements were also assessed. Psychological distress was measured using the total score of the K-6, which assesses a spectrum of mental distress, including symptoms commonly associated with anxiety disorders, depressive disorders, and general psychological distress (32). The K-6 includes six items evaluating non-specific symptoms of distress such as feeling hopeless, nervous, restless, or overwhelmed, providing a robust measure of psychological wellbeing over the past month. The total score ranges from 0 to 24, with higher scores indicating greater levels of distress. The validity and reliability of the K-6 have been well-established in previous research (33).

### 2.4 Statistical analysis

All statistical analyses were performed using two-sided tests, carried out by SPSS software version 27 (IBM Corporation, Armonk, New York, United States) and AMOS version 22. Two-sample independent t and chi-squared tests were used to test for differences in demographic characteristics of the sample (age, education level, and BMI etc.), IE and MEQ scores, and mental distress levels among genders; Cohen's d effect size coefficients were calculated to quantify the magnitude of differences observed in these variables. Effect sizes were interpreted as small (0.2 ≤ d < 0.5), medium (0.5 ≤ d < 0.8), or large (d ≥ 0.8) according to Cohen's guidelines (34).

**Figure 2:**
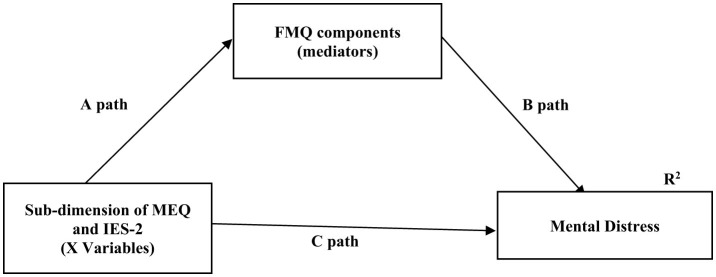
Hypothesized model in which FMQ components as a mediating variable sub-dimension of IES-2 and MEQ to mental distress. FMQ, Food mood questionnaire; IES, Intuitive eating scale; MEQ, Mindful eating questionnaire. Data are standardized regression weights (β) for paths. A, B, and C paths expresses the direct effects of the independent variable on the dependent variable at the location. C^¶^ (indirect effects) = A path × B path. Total effects = C + C^†^. R^2^ = Coefficient of explanation.

Multicollinearity among predictors (food intake and exercise) was assessed using Variance Inflation Factor (VIF) values, with no predictors exceeding 10, and the correlation matrix revealed no coefficients above 0.8. Structural equation modeling (SEM) was applied to assess the proposed theoretical models. Conceptual models were first developed based on the literature, and the hypothesized models—where FMQ components serve as mediators between IE, MEQ, and mental distress—are summarized in Figure 2. Pathway “A” determined the regression coefficients for the effect of the independent variable (X) on FMQ components (mediators). Pathway B examined the association between mediator and mental distress. Pathway C measured the direct effect of X on mental distress. Pathway C^¶^ examined the indirect effect of IE or MEQ on dependent variable through FMQ components. Before testing the hypotheses, a bivariate correlation was run to analyze the associations between IE, MEQ, food and mental distress variables. According to the correlation matrix output, they were included in the SEM if the correlation relationship with dependent and independent variables was >0.30 (moderate association). SEM is a combination of two parts, measurement model (latent variables identified using factor analysis), which was not applicable for present study, and the structural model (direct and indirect pathways of associations between latent and other observed variables). Standardized regression coefficients were estimated using the Maximum Likelihood Estimation Procedure (ML). The total, direct, and indirect effects, 95% confidence intervals, and explanation coefficients were presented in the SEM analysis. Bootstrapping (5,000 replications) was used to generate normal-based bootstrapped confidence intervals around the indirect effect. If the total effect is statistically significant and the confidence interval of the indirect effect does not include zero, then there is a mediation effect. Partial mediation occurs if the direct effect is statistically significant; otherwise, full or complete mediation. To verify the fit of the model, some measurements were analyzed: chi-square fit statistics/degree of freedom (CMIN/DF) value <2, indicating a reasonable fit; root mean square error of approximation (RMSEA) and standardized root mean square residual (SRMR), with values <0.08 and <0.10, respectively indicating that the theoretical model fits the data; Tucker–Lewis index (TLI) >0.95 and comparative fit index (CFI) >0.95 and goodness-of-fit statistic (GFI), where values >0.90 indicate a good fit of the model. *P*-values < 0.05 were considered statistically significant (34).

## 3 Results

### 3.1 Participant characteristics

Table 1 provides an overview of the participants' general characteristics, stratified by gender, along with their IE, MEQ, and K-6 mental distress scores. The mean age of the participants was 30.4 ± 11.5 years and male participants were higher than female participants (*p* < 0.001). The distribution of chronic disease status, drug use and MEQ scores was found to be similar in female and male participants. While the mean BMI of males was slightly higher than that of females, the effect size was negligible (Cohen's d effect size = 0.19). Men had higher IE scores and sub-dimensions compared to females except for the B-FCC, but the effect sizes of these differences were small or negligible. In addition, male participants had lower mental distress scores than female participants, and the effect size was small (Cohen's d effect size= 0.27).

**Table 1:** Comparison of participants' general characteristics, intuitive, and mindful eating and mental distress by gender.

	**Men (*n =* 273)**	**Women (*n =* 717)**	**Total (*n =* 990)**	***p***	**Cohen's d Effect size**
Age (yrs)	34.2 ± 12.2	28.9 ± 10.9	30.4 ± 11.5	<0.001^**^	0.46
**Education level**					
Primary	41 (15.0%)	106 (14.8%)	147 (14.8%)		
High school	74 (27.1%)	97 (13.5%)	171 (17.3%)	<0.001^**^	0.83
Associate degree	47 (17.2%)	121 (16.9%)	168 (17.0%)		
Undergraduate	111 (40.7%)	393 (54.8%)	504 (50.9%)		
**Smoking status**					
Current	92 (33.7%)	104 (14.5%)	196 (19.8%)		
Never	152 (55.7%)	585 (81.6%)	737 (74.4%)	<0.001^**^	0.18
Former	29 (10.6%)	28 (3.9%)	57 (5.8%)		
**Chronic disease**					
No	214 (78.4%)	593 (82.8%)	807 (81.5%)	0.091	0.03
Yes	59 (21.6%)	124 (17.2%)	183 (18.4%)		
**Drug use**					
No	238 (87.2%)	641 (89.4%)	879 (88.7%)	0.137	0.01
Yes	35 (12.8%)	76 (10.6%)	111 (11.3%)		
BMI (kg/m^2^)	24.4 ± 4.5	25.3 ± 4.1	24.7 ± 4.5	0.006^*^	0.19
IES total	14.2 ± 2.0	13.4 ± 2.1	13.6 ± 2.1	<0.001^**^	0.35
UPE	3.3 ± 0.5	3.2 ± 0.5	3.3 ± 0.5	<0.001^**^	0.32
EPR	3.5 ± 0.6	3.2 ± 0.6	3.3 ± 0.6	<0.001^**^	0.42
RHSC	3.6 ± 0.5	3.3 ± 0.6	3.4 ± 0.6	<0.001^**^	0.12
B-FCC	3.7 ± 0.9	3.6 ± 0.9	3.6 ± 0.9	0.079	0
MEQ total	5.4 ± 0.6	5.5 ± 0.6	5.5 ± 0.6	0.121	0.13
Awareness	2.6 ± 0.5	2.7 ± 0.5	2.7 ± 0.5	0.061	0.19
Disinhibition	2.8 ± 0.5	2.8 ± 0.5	2.8 ± 0.5	0.248	0.02
Mental distress	9.1 ± 5.2	11.7 ± 5.2	11.0 ± 5.3	<0.001^**^	0.27

B-FCC, Body-food choice congruence; BMI, Body mass index; EPR, Eating for physical rather than emotional reasons; IES, Intuitive eating scale; MEQ, Mindful eating questionnaire; RHSC, Reliance on hunger and satiety cues; UPE, Unconditional permission to eat. Participant characteristics stratified by gender, including demographics, intuitive eating (IES-2), mindful eating (MEQ), and mental distress scores (K-6). Numeric data presented as mean ± standard deviation; categorical data presented as frequency (%). Statistical comparisons performed using independent *t-*test and Chi-square test.

* *p* < 0.01,

** *p* < 0.001.

### 3.2 Correlation matrix analysis

In the correlation matrix, both IE and ME demonstrated moderate negative associations with mental distress (r = −0.477 and −0.361, respectively; *p* < 0.001). Mental distress was moderately negatively associated with breakfast, dairy products, fruit, nuts, and DGLV but weakly associated with exercise. Moreover, IE and ME scores were moderately positively associated with exercise, breakfast, dairy, fruits, nuts, and DGLV (Table 2). To ensure both statistical robustness and clinical relevance, only FMQ components exhibiting moderate or stronger correlations (r ≥ 0.30) with mental distress and IE/ME scales were included in the SEM. Components with r < 0.30 failed to produce statistically significant pathways in preliminary SEM and explained minimal variance in key outcomes, indicating limited clinical importance; therefore, they were excluded from the final mediation model. This approach provided a clear, data-driven basis for variable selection in our mediation analyses.

**Table 2:** Correlation matrix output between intuitive eating, mindful eating, and FMQ.

**Variables**	**1**.	**2**.	**3**.	**4**.	**5**.	**6**.	**7**.	**8**.	**9**.	**10**.	**11**.	**12**.	**13**.	**14**.	**15**.	**16**.	**17**.
**1. Mental Distress**	-																
**2.IE**	−0.477^*^	-															
**3.MEQ**	−0.361^*^	0.413^*^	-														
**4.Exercise**	−0.301^*^	0.322^*^	0.352^*^	-													
**5.Breakfast**	−0.400^*^	0.350^*^	0.378^*^	0.240^*^	-												
**6.Whole Grain**	−0.113^*^	0.055	0.060	0.133^*^	0.171^*^	-											
**7.Dairy**	−0.431^*^	0.445^*^	0.315^*^	0.252^*^	0.424^*^	0.159^*^	-										
**8.Caffeine**	0.059	0.018	−0.001	−0.063	0.082	0.074	0.108^*^	-									
**9.Fruits**	−0.475^*^	0.381^*^	0.356^*^	0.258^*^	0.360^*^	0.142^*^	0.396^*^	0.059	-								
**10.Nuts**	−0.196^*^	0.139^*^	0.153^*^	0.170^*^	0.242^*^	0.270^*^	0.225^*^	0.008	0.371^*^	-							
**11.HGI food**	0.019	−0.025	0.006	0.043	0.055	0.126^*^	0.014	0.058	0.092	0.119^*^	-						
**12.Meat**	−0.075	0.046	0.102^*^	0.092	0.163^*^	0.176^*^	0.091	0.076	0.191^*^	0.299^*^	0.290^*^	-					
**13.DGLV**	−0.478^*^	0.392^*^	0.363^*^	0.300^*^	0.360^*^	0.206^*^	0.380^*^	−0.008	0.477^*^	0.340^*^	0.084	0.223^*^	-				
**14.Beans**	−0.112^*^	0.069^*^	0.101^*^	0.102^*^	0.128^*^	0.193^*^	0.082	−0.056	0.160^*^	0.255^*^	0.259^*^	0.181^*^	0.330^*^	-			
**15.Seafood**	−0.180^*^	−0.001	−0.024	0.130^*^	0.083	0.067	0.053	−0.107^*^	0.115^*^	0.259^*^	0.072	0.093	0.211^*^	0.296^*^	-		
**16.Fast food**	0.179^*^	−0.165^*^	−0.104^*^	−0.042	−0.150^*^	−0.020	−0.183^*^	−0.013	−0.129^*^	0.053	0.257^*^	0.135^*^	−0.063	0.205^*^	0.108^*^	-	
**17.Sugary food**	0.166^*^	−0.082	−0.003	−0.088	−0.060	−0.029	−0.068	0.164^*^	−0.018	0.000	0.229^*^	0.092	−0.100^*^	−0.010	−0.080	0.304^*^	-

Correlation matrix illustrating relationships between intuitive eating (IES), mindful eating (MEQ), Food-Mood Questionnaire (FMQ) components, and mental distress. DGLV, Dark green leafy vegetables; HGI, High glycemic index; IES, Intuitive eating scale; MEQ, Mindful eating questionnaire.

* *p* < 0.001.

### 3.3 Path analysis of FMQ components between total MEQ and IE score and mental distress

**Figure 3:**
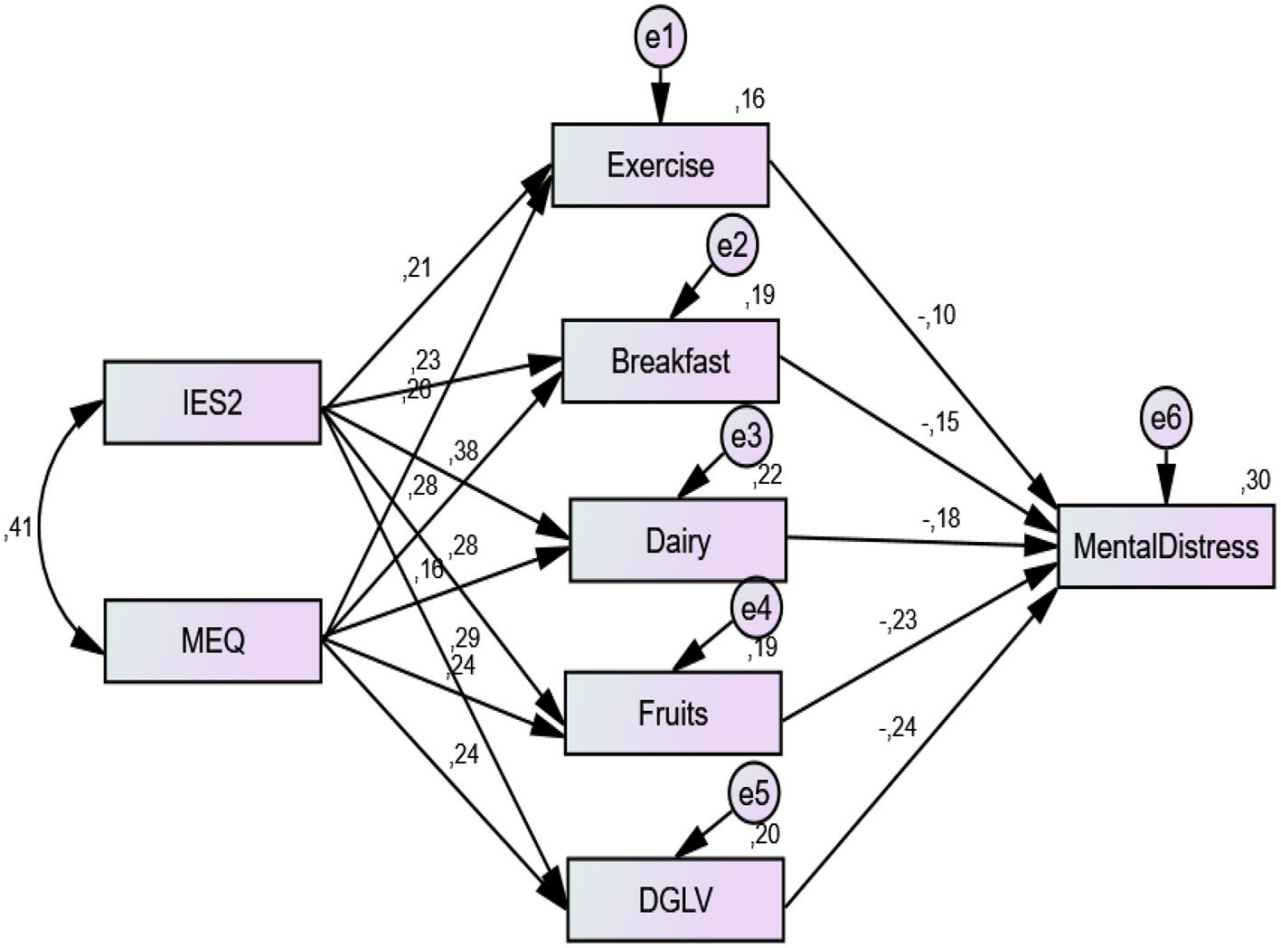
Path analysis of IES-2 and MEQ on mental distress in combination with foods and exercise. Structural equation model illustrating pathways linking intuitive eating (IES-2 total score), mindful eating (MEQ total score), dietary factors (exercise, breakfast, dairy, fruits, dark green leafy vegetables [DGLV]), and mental distress. Standardized regression coefficients and error terms (e) are shown. All regression path coefficients were statistically significant. Model fit indices: χ^2^/df = 1.414, GFI = 0.928, CFI = 0.956, TLI = 0.953, RMSEA = 0.083, SRMR = 0.079.

The direct and indirect effects of ME, IE, and specific FMQ components on mental distress levels are presented in Figure 3 and Table 3. Notably, dark green leafy vegetables (DGLV) and fruits were the strongest predictors of reduced mental distress, with standardized regression coefficients of −0.238 and −0.235, respectively, emphasizing their prominent role in psychological wellbeing. The association between MEQ scores and mental distress was mediated through their positive relationship with higher dairy and fruit intake (β = 0.376 and 0.274, respectively). IES-2 and MEQ explained 16% of exercise, 19% of breakfast consumption, 22% of dairy intake, and 19% and 20% of fruit and DGLV intake, respectively. Collectively, all independent variables accounted for 30% of the variance in mental distress, with the model demonstrating strong fit indices (χ^2^/df = 1.414, GFI = 0.928, CFI = 0.956, TLI = 0.953, RMSEA = 0.083, SRMR = 0.079). Additionally, the indirect associations of IES-2 and MEQ with mental distress were statistically significant and mediated through FMQ sub-dimensions (−0.262 and −0.212, respectively), which explained 65.7% of the variance in mental distress levels. The remaining 34.3% was attributed to direct pathways involving IES-2 and MEQ (Table 3).

**Table 3:** The total, direct and indirect effect of mindful and intuitive eating, foods and exercise on mental distress.

**Variables**	**Direct**	**Indirect**
IES	-	−0.262
MEQ	-	−0.212
Exercise	−0.104	-
Breakfast	−0.151	-
Dairy	−0.181	-
Fruits	−0.235	-
DGLV	−0.238	-
Through total causal effect^£^	−0.909	−0.474
Percentage of direct and indirect effects	−0.909/[−0.909 + (−0.474)] = 65.7%	−0.474/[−0.909 + (−0.474)] = 34.3%

Overall direct and indirect associations of intuitive eating scale total score (IES), mindful eating questionnaire total score (MEQ), and selected dietary and lifestyle practices (exercise, breakfast, dairy, fruits, dark green leafy vegetables [DGLV]) with mental distress. Standardized regression coefficients are presented. DGLV, Dark green leafy vegetables; HGI, High glycemic index; IES, Intuitive eating scale; MEQ, Mindful eating questionnaire.^£^Total effect is defined as the sum of direct and indirect effects.

### 3.4 SEM analysis of IE and ME sub-dimensions, mental distress and FMQ components

The correlation matrix relationships between IE and ME sub-dimensions, food mood components, and mental distress scores are detailed in Supplementary Table 1. Variables with correlation coefficients ≥0.30 were included in the structural equation model (Table 4). Table 3 summarizes the general direct and indirect associations among IE, ME, FMQ components, and mental distress, while Table 4 specifically examines these associations for each IE sub-dimension separately. The direct effects of most IE sub-dimensions on mental distress were significant, except for UPE and B-FCC. The negative associations between UPE, RHSC, and mental distress were mediated through pathways involving exercise, breakfast, dairy, fruits, and DGLV. Notably, dairy consumption fully mediated the relationship between mental distress and UPE (β = −0.100 [95% CI: −0.128 to −0.076]) and B-FCC (β = −0.119 [95% CI: −0.149 to −0.093]). The negative effects of disinhibition on mental distress were partially mediated through breakfast and DGLV. The explanatory power (R^2^) of IE and ME sub-dimensions and FMQ components for mental distress ranged from 0.219 to 0.338. Fruits (R^2^ = 0.338) and DGLV (R^2^ = 0.313) had the highest predictive coefficients for mental distress levels. Full goodness-of-fit indices for SEM models are presented in Supplementary Table 2, confirming the adequacy of the model.

**Table 4:** Direct and indirect effects of IES-2 and MEQ sub-dimensions and FMQ components on mental distress among all individuals using SEM.

**Relationship**	**Mediators**	**Direct effect of X on Mental distress**	**Association between X and mediators**	**Mediation or Indirect effect**	**Coefficient of explanation**	**Mediation effect**
		**C path (SE)**	**A path (SE)**	**C** ^¶^ **path (95% CI)**	**R** ^2^	
EPR 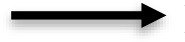 Mental Distress	Exercise	−0.388 (0.027)	0.275 (0.031)	−0.053 [−0.077– (−0.035)]	0.230	**Partial**
	Breakfast	−0.345 (0.028)	0.340 (0.029)	−0.096 [−0.123– (−0.073)]	0.266	**Partial**
	Dairy	−0.317 (0.032)	0.419 (0.029)	−0.125[−0.158– (−0.098)]	0.282	**Partial**
	Fruit	−0.322 (0.019)	0.325 (0.021)	−0.120[−0.148– (−0.095)]	0.338	**Partial**
	DGLV	−0.310 (0.025)	0.357 (0.029)	−0.131[−0.160– (−0.105)]	0.313	**Partial**
UPE 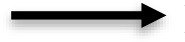 Mental distress	Dairy	−0.191 (0.029)	0.263 (0.027)	−0.100[−0.128– (−0.076)]	0.220	**Full**
RHSC 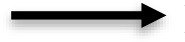 Mental distress	Exercise	−0.378 (0.032)	0.318 (0.030)	−0.058[−0.083– (−0.037)]	0.219	**Partial**
	Breakfast	−0.339 (0.030)	−0.339 (0.031)	−0.097[−0.124– (−0.075)]	0.263	**Partial**
	Dairy	−0.316 (0.032)	0.387 (0.027)	−0.119[−0.150– (−0.094)]	0.271	**Partial**
	Fruit	−0.310 (0.031)	0.340 (0.028)	−0.126[−0.154– (−0.100)]	0.310	**Partial**
	DGLV	−0.298 (0.031)	0.375 (0.027)	−0.137[−0.112– (−0.166)]	0.305	**Partial**
B-FCC 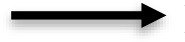 Mental distress	Dairy	−0.229 (0.030)	0.338 (0.029)	−0.119[−0.093– (−0.149)]	0.232	**Full**
	Fruits	−0.224 (0.029)	0.307 (0.028)	−0.125[−0.099 (−0.154)]	0.272	**Full**
Disinhibition. 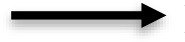 Mental distress	Breakfast	−0.315 (0.026)	0.316 (0.031)	−0.095[−0.072– (−0.122)]	0.249	**Partial**
	DGLV	−0.289 (0.027)	0.311 (0.029)	−0.121[−0.093– (−0.149)]	0.304	**Partial**

B-FCC, Body-food choice congruence; DGLV, Dark green leafy vegetables; EPR, Eating for physical rather than emotional reasons; RHSC, Reliance on hunger and satiety cues; UPE, Unconditional permission to eat. Detailed direct and indirect associations between intuitive eating scale (IES-2) sub-dimensions (Eating for Physical rather than Emotional Reasons [EPR], Unconditional Permission to Eat [UPE], Reliance on Hunger and Satiety Cues [RHSC], Body-Food Choice Congruence [B-FCC]) and mental distress, mediated by specific dietary and lifestyle components. Standardized regression coefficients (C path, A path, C' path), 95% confidence intervals, explained variance (R^2^), and mediation type (partial/full) are reported. Partial mediation means that the mediating variable explains part of the relationship between the independent and dependent variables, while full mediation means that the mediating variable fully explains this relationship and eliminates the direct effect. All standardized path coefficients and standardized residual covariance shown were significant except for the direct effect between UPE and B-FCC to mental stress.

## 4 Discussion

Although there is a substantial body of literature suggesting that IE or ME may have an impact on mental health (35, 36), there is no research on the mediation of dietary patterns in the association of IE and ME on mental distress. To our knowledge, this study is the first to assess these outcomes in a general population. The study revealed that individuals who scored high on IE and ME showed healthier habits as identified by the five subcomponents of the FMQ. Furthermore, individuals who consumed breakfast, dairy, DGLV, fruit, and nuts more frequently also had significantly lower mental distress. The practice of IE and ME was also shown to be positively associated with mental wellbeing. These effects are mediated by diet and exercise.

The present study aligns with existing literature demonstrating that Intuitive Eating (IE) is associated with healthier dietary patterns and lower mental distress. Individuals with higher IE scores reported greater consumption of nutrient-dense foods such as dairy, fruits, and dark green leafy vegetables (DGLV), consistent with findings by Christoph et al., who observed similar dietary patterns across genders (16). This suggests that IE fosters a food selection process guided by internal hunger and satiety cues rather than external restrictions, leading to more balanced nutritional intake.

Notably, IE was inversely correlated with mental distress, reinforcing prior research linking it to reduced depressive symptoms, anxiety, and negative affect (16, 37, 38). The Austrian rural population study (26) and a recent meta-analysis (38) (r= −0.34 for anxiety, r= −0.29 for depression) support our findings, highlighting IE's protective role in psychological wellbeing. By rejecting rigid dieting rules and promoting body trust, IE may mitigate.

However, findings regarding the Unconditional Permission to Eat (UPE) subscale of Intuitive Eating (IE) have been inconsistent across studies. For instance, Camilleri et al. found that higher UPE scores were associated with lower intakes of fruits, vegetables, and whole grains, and higher consumption of sweets and fats, particularly among both women and men (14). Similarly, Horwath et al. reported small but significant associations between higher UPE scores and decreased consumption of nutrient-dense foods, alongside increased intake of sweets, fast food, and salty snacks (11). In the present study, individuals with higher UPE scores were found to consume significantly less dairy; however, no significant associations were observed between UPE and the consumption of energy-dense foods such as fast food or sweets, as measured by the FMQ. While the FMQ includes several items that could be considered indicative of permissive eating behaviors—such as high-glycaemic index foods, fast food, and sugary items—these components did not exhibit strong enough associations with either UPE or mental distress to be retained in the final mediation model. Therefore, although our findings do not support a direct link between UPE and indulgent or high-calorie dietary choices, this may reflect limitations in the FMQ's ability to capture the broader construct of dietary permissiveness, including portion size, snacking frequency, and subjective perceptions of food autonomy. We acknowledge this as a limitation of our dietary assessment approach and recommend that future research employ more comprehensive tools to assess permissive or hedonic eating patterns in relation to UPE. Additionally, the variability in findings across studies may, in part, be attributable to differences in sample characteristics and study design, such as the use of larger cohort samples. Further investigation is warranted to elucidate the psychological mechanisms underlying the heterogeneous effects of UPE on diet quality.

Higher RHSC scores were found to be associated with exercise and greater healthy food choices such as breakfast, dairy, fruits, and DGLV. On the other hand, in large French study, higher RHSC levels were found to be associated with higher whole grain intake among women but not among men (14). The RHSC was found to be associated with lower fruit and vegetable consumption among male university students but not among females (15). Notably, this study observed similar results between men and women, which differs from previous studies (11, 14, 15). Although the findings of different studies do not always align, the results of this study indicate that IE is moderately associated with some markers of improved dietary intake but not consistently associated with all markers of a healthy diet when considered in the context of previous research.

Crucially, our study is among the first to identify dietary patterns as mediators in the IE–mental health relationship. The present study has demonstrated that IE is associated with a reduction in mental distress through the consumption of healthy foods, including dairy products, exercise, DGLV and nuts, and exercise. A diet comprising nutritionally dense foods and regular exercise has been demonstrated to enhance wellbeing by increasing dopamine levels in the brain. The findings of this study indicate that a diet comprising breakfast, dairy products, exercise, and DGLV consumption may play an essential role in fostering a positive cycle. Dairy products are a significant source of magnesium and protein, both essential for mental health (39). Previous studies have demonstrated a protective effect of dairy consumption on mental health (40–42). It can be posited that dairy products can support adequate calcium intake, given that they are a good source of calcium (43). Adequate calcium intake may be considered a potential intervention to improve mental health, given its biological functions in the nervous system (44). Calcium is a key regulator of neurotransmitter synthesis and release, which are essential for neuronal activation and mood regulation (45). Furthermore, calcium is necessary for the synthesis of serotonin, the precursor of melatonin (46). Melatonin is a key regulator of sleep cycles, and sleep plays a fundamental role in maintaining emotional health (47). Given that dairy products provide a good or excellent source of bioavailable calcium, the promotion of the consumption of dairy products may improve certain indicators of mental health (48).

ME similarly correlated with reduced mental distress but operated through slightly different mechanisms compared to IE. While IE emphasizes responsiveness to internal bodily cues—such as hunger and satiety—ME is grounded in the cultivation of present-moment awareness and attentional control during eating experiences. This structured and deliberate approach may explain ME's independent association with improved mental wellbeing, even after controlling for dietary mediation (9). ME engages cognitive processes such as mindfulness meditation and body scanning, which enhance emotional regulation and reduce impulsivity around food (49). These practices help decouple eating behaviors from emotional triggers and foster reflective, non-judgmental awareness, contributing directly to psychological resilience. A growing body of evidence supports the efficacy of mindfulness-based interventions in mitigating symptoms of depression, stress, and disordered eating (50–52). For example, a study among nurses found that those with higher ME scores also reported greater mental wellbeing (53). Similarly, the present findings show that higher MEQ scores are associated with reduced mental distress. ME's emphasis on attentional control may also promote healthier dietary choices—such as regular breakfast and dairy intake—by reducing susceptibility to emotional or impulsive eating. In contrast, while IE facilitates a healthier relationship with food through internal attunement and reduced dietary restraint, it lacks the structured attentional strategies that characterize ME. Thus, while both ME and IE support psychological wellbeing, they do so via distinct cognitive and behavioral pathways: IE through intuitive, physiological alignment and ME through deliberate, mindful engagement with the eating experience.

This is because individuals with highly ME habits are known to make healthier food choices, such as those involving the consumption of dairy products and having breakfast, and healthy food choices are known to cause lower mental distress. Furthermore, although diet does not have a mediating association, the possession of ME and eating wisdom directly indicates that the individual has superior mental health.

The integration of IE and ME into one's lifestyle is associated with increased adoption of healthier eating habits and an active lifestyle, which in turn is linked to better overall mental wellbeing (54). Our findings indicate that associations between breakfast consumption, dairy intake, exercise, and DGLV consumption and improvements in mental wellbeing were observed. The combination of these nutrients may be associated with enhanced mood and cognitive function (39). Collectively, these observations suggest that green leafy vegetable and fruit consumption are associated with certain mental health indicators. Green leafy vegetables and fruits contain potent antioxidant phytochemicals, including anthocyanins, flavonoids, carotenoids, and flavonols (55). A significant number of these biochemical compounds have been demonstrated to enhance brain function by increasing levels of brain-derived neurotrophic factor (BDNF), which has anti-inflammatory and anti-apoptotic properties (56). Similarly, exercise has been shown to be associated with increased BDNF release, which is linked to improved brain function and mental wellbeing (57). The development of these functions may in turn be associated with positive shifts in mood. Together, these mechanisms—including antioxidative and neurotrophic effects of phytochemicals, calcium-dependent regulation of neurotransmitter synthesis, and serotonin modulation—may collectively underpin the observed associations between IE/ME-related behaviors and improved mental wellbeing.

### 4.1 Strengths of the study

This study's strengths include the large random population sample, the inclusion of both males and females, and the fact that all four IE dimensions were analyzed. The majority of research into IE has been conducted with female participants (26). Moreover, numerous studies utilizing the IES have been conducted with samples of students (10) or community groups (58) and have collected data through print advertisements or online surveys (59). Another strength of the study is the use of self-administered scales with established validity and reliability, as well as the face-to-face collection of data.

### 4.2 Limitations of the study

Although this study provided valuable insights into the impact of IE and dietary patterns on mental distress, it also had some limitations. Firstly, the study's cross-sectional nature means that it is only possible to suggest the potential relationships between the variables examined rather than proving causal relationships. Secondly, although the use of validated FMQ, IES-2, or MEQ questionnaires, which have previously been examined among the Turkish population, was employed, it is acknowledged that they are susceptible to potential measurement errors, misclassification of foods, and psychological distress cases. Additionally, despite measures to minimize social desirability bias, self-reported data inherently carry limitations, including potential underreporting or overreporting due to participants' desire to present themselves positively, inaccuracies in recall of dietary intake or behaviors, and varying individual interpretations of questionnaire items. Cultural and geographic factors have been demonstrated to influence dietary preferences and practices, which in turn have been linked to mental health outcomes (60). Geographic factors, such as urban vs. rural living, affect access to healthy food options and exercise facilities, influencing dietary habits and mental health (61). In urban Turkish settings, ongoing nutrition transition—with increased availability and consumption of processed and fast foods, sugar-sweetened beverages, and convenience meals—may challenge the practice of IE and ME by promoting external food cues and habitual consumption patterns. Conversely, rural populations often adhere more closely to traditional dietary practices, characterized by home-cooked meals and seasonal produce, which may facilitate stronger attunement to internal hunger and satiety signals and more mindful food experiences. A variety of factors have been demonstrated to influence the cultural composition of individuals, including religion, cultural beliefs, and the nature of community and regional living. These factors have been shown to impact mental distress through the medium of food choice in diverse populations (62). The findings cannot be extrapolated to the entire Turkish population, as the sample is drawn from a single city in Türkiye. It is important to note that despite our efforts to control for numerous potential confounding factors, including BMI and socioeconomic disparities, it is not possible to eliminate the possibility of residual confounding. Therefore, a cautious interpretation of the results is recommended. Finally, our assessment of physical activity relied on a single self-reported item, which may not capture the multidimensional nature of exercise. Future studies should consider using validated multi-item instruments (e.g., the International Physical Activity Questionnaire) or objective tools such as accelerometers to measure frequency, intensity, duration, and type of physical activity more comprehensively.

## 5 Conclusion

The present study demonstrates a significant positive correlation between adherence to Intuitive Eating (IE) and Mindful Eating (ME) practices and healthier dietary and lifestyle behaviors, as well as enhanced psychological wellbeing. Also, intuitive dietary approaches that rely on physical reasons or hunger and satiety cues rather than extrinsic factors may be associated with reduced psychological stress through greater consumption of breakfast, dairy products, fruit, and dark green leafy vegetables. This study provides robust cross-sectional evidence that IE and ME influence mental distress via positive lifestyle factors, such as healthy dietary patterns and regular exercise, which collectively reduce mental distress. Further longitudinal experimental studies are required to provide conclusive evidence of these relationships. Researchers may consider incorporating IE and ME assessment tools into psycho-nutritional interventions, pending validation through future longitudinal research.

## Data Availability

The raw data supporting the conclusions of this article will be made available by the authors, without undue reservation.
