# Supplementary material for: Links between intuitive and mindful eating and mood: do food intake and exercise mediate this association?
Source: Front Nutr. 2025 May 30;12:1458082. doi: 10.3389/fnut.2025.1458082 (PMC12162310; doi:10.3389/fnut.2025.1458082)
Supplement: Supplementary file 1 [file Table_1.docx]

**SUPPLEMANT FILE**

**Supplemantary Table 1.** Correlation matrix output between sub-dimension of intuitive mindful eating and FMQ components

| Variables | 1. | 2. | 3. | 4. | 5. | 6. | 7. | 8. | 9. | 10. | 11. | 12. |
| --- | --- | --- | --- | --- | --- | --- | --- | --- | --- | --- | --- | --- |
| 1.Mental distress | - |  |  |  |  |  |  |  |  |  |  |  |
| 2.Awareness | -.006 | - |  |  |  |  |  |  |  |  |  |  |
| 3.Disinhibition | -.410^*^ | -.285^*^ | - |  |  |  |  |  |  |  |  |  |
| 4.UPE | -.312^*^ | .011 | .260^*^ | - |  |  |  |  |  |  |  |  |
| 5.EPR | -.442^*^ | -.009 | .427^*^ | .600^*^ | - |  |  |  |  |  |  |  |
| 6.RHSC | -.435^*^ | .025 | .377^*^ | .385^*^ | .602^*^ | - |  |  |  |  |  |  |
| 7.B-FCC | -.348^*^ | .037 | .363^*^ | .426^*^ | .506^*^ | .506^*^ | - |  |  |  |  |  |
| 8.Exercise | -.301^*^ | .156^*^ | .262^*^ | .183^*^ | .305^*^ | .318^*^ | .244^*^ | - |  |  |  |  |
| 9.Breakfast | -.400^*^ | .129^*^ | .316^*^ | .203^*^ | .340^*^ | .339^*^ | .238^*^ | .240^*^ | - |  |  |  |
| 10.Dairy | -.431^*^ | .086^*^ | .283^*^ | .331^*^ | .419^*^ | .387^*^ | .338^*^ | .252^*^ | .424^*^ | - |  |  |
| 11.Fruits | -.475^*^ | .136^*^ | .284^*^ | .225^*^ | .325^*^ | .340^*^ | .307^*^ | .258^*^ | .360^*^ | .396^*^ | - |  |
| 12.DGLV | -.478^*^ | .116^*^ | .311^*^ | .236^*^ | .358^*^ | .375^*^ | .281^*^ | .300^*^ | .360^*^ | .380^*^ | .477^*^ | - |

B-FCC = Body-food choice congruence; DGLV= Dark green leafy vegetables; EPR = Eating for physical rather than emotional reasons; RHSC = Reliance on hunger and satiety cues; UPE = Unconditional permission to eat.

^*^p<0.001

**Supplemantary Table 2.** Goodness of fit indices for structual equation models

| Relationship | Mediators | χ2/df  (<2.00) | GFI  (>0.90) | CFI  (>0.95) | TLI  (>0.95) | RMSEA  (<0.08) | SRMR  (<0.10) |
| --- | --- | --- | --- | --- | --- | --- | --- |
| EPC Mental Distress | Exercise | 0.482 | 1.000 | 0.994 | 1.000 | 0.035 | 0.022 |
|  | Breakfast | 0.000 | 0.998 | 1.000 | 0.962 | 0.027 | 0.001 |
|  | Dairy | 0.683 | 0.961 | 0.972 | 1.000 | 0.049 | 0.035 |
|  | Fruits | 0.162 | 1.000 | 1.000 | 0.974 | 0.045 | 0.013 |
|  | DGLV | 0.505 | 0.972 | 0.984 | 0.951 | 0.039 | 0.029 |
| UPE Mental Distress | Dairy | 1.315 | 0.912 | 1.000 | 0.986 | 0.032 | 0.017 |
| RHSC Mental Distress | Exercise | 0.350 | 0.997 | 1.000 | 0.984 | 0.032 | 0.002 |
|  | Breakfast | 0.140 | 0.964 | 1.000 | 1.000 | 0.037 | 0.009 |
|  | Dairy | 0.577 | 0.921 | 0.951 | 1.000 | 0.062 | 0.072 |
|  | Fruit | 1.488 | 1.000 | 1.000 | 0.969 | 0.045 | 0.083 |
|  | DGLV | 1.509 | 0.943 | 1.000 | 0.972 | 0.041 | 0.077 |
| B-FCC Mental Distress | Dairy | 1.342 | 0.917 | 0.965 | 1.000 | 0.036 | 0.052 |
|  | Fruits | 1.410 | 0.937 | 0.958 | 1,000 | 0.037 | 0.041 |
| Disinhibition Mental Distress | Breakfast | 1.388 | 1.000 | 1.000 | 0.984 | 0.029 | 0.060 |
|  | DGLV | 0.458 | 0.945 | 0.964 | 1.000 | 0.037 | 0.021 |

χ2/df= Chi-Square value/Degrees of freedom; CFI= Comparative fit index; GFI= goodness-of-fit index; RMSEA= Root mean square error of approximation; SRMR= Standardized root mean square residual; TLI= Tucker–Lewis index.

It shows the goodness-of-fit values for the SEM analyses in Table 4. All goodness-of-fit values for each model were at acceptable levels. Therefore, modification index for models was not required.
